# The Human Cytomegalovirus Major Immediate-Early Proteins as Antagonists of Intrinsic and Innate Antiviral Host Responses

**DOI:** 10.3390/v1030760

**Published:** 2009-11-05

**Authors:** Christina Paulus, Michael Nevels

**Affiliations:** Institute for Medical Microbiology and Hygiene, University of Regensburg, Franz-Josef-Strauss-Allee 11, D-93053 Regensburg, Germany; E-Mail: christina.paulus@klinik.uni-regensburg.de

**Keywords:** cytomegalovirus, CMV, innate immunity, intrinsic defense, interferon response, nuclear domain 10, apoptosis, immediate-early genes, IE1, IE2

## Abstract

The major immediate-early (IE) gene of human cytomegalovirus (CMV) is believed to have a decisive role in acute infection and its activity is an important indicator of viral reactivation from latency. Although a variety of gene products are expressed from this region, the 72-kDa IE1 and the 86-kDa IE2 nuclear phosphoproteins are the most abundant and important. Both proteins have long been recognized as promiscuous transcriptional regulators. More recently, a critical role of the IE1 and IE2 proteins in counteracting non-adaptive host cell defense mechanisms has been revealed. In this review we will briefly summarize the available literature on IE1- and IE2-dependent mechanisms contributing to CMV evasion from intrinsic and innate immune responses.

## 1. Human Cytomegalovirus (CMV) Is a Significant Pathogen

CMV, the prototype β-herpesvirus, is the cause of a “silent pandemic” that continuously inflicts suffering upon people including immunocompromised patients as well as pregnant mothers and their unborn or prematurely newborn babies (reviewed in [1]). In the absence of an approved vaccine viral DNA polymerase inhibitors, including the nucleoside analogon ganciclovir, have provided major advances in CMV disease management. However, use of these drugs is limited by significant toxicity and relatively modest effectiveness due to poor bioavailability and problems with viral drug resistance (reviewed in [2]). Furthermore, no drug has been licensed to treat congenital CMV infection, and recent trials with newly developed antivirals targeting CMV have not lived up to their expectations. Thus, it is still imperative to develop new anti-CMV strategies directed at appropriate viral molecular targets.

## 2. The CMV Major Immediate-Early (IE) Proteins Are Multifunctional Key Regulators

### 2.1. Structure and Importance of the Major IE Gene

**Figure 1.**
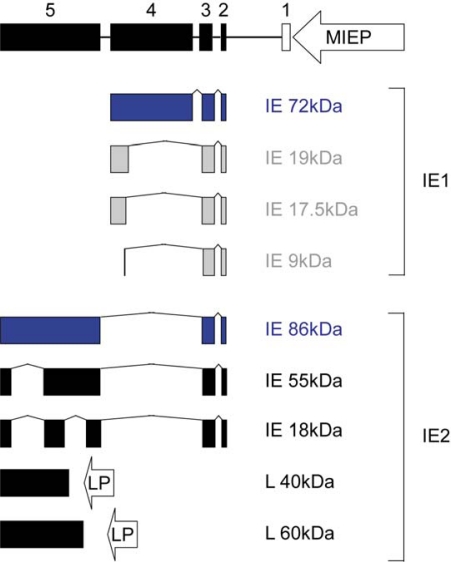
Structural organization and protein products of the CMV major IE locus. At the top of the diagram, the lengths and relative positions of exons 1 to 5 (the four coding exons are presented as black boxes and non-coding exon 1 as open box) and the location of the major IE promoter-enhancer (MIEP) are shown. Proteins are subdivided into the IE1 (containing exon 4 sequences) and IE2 (containing exon 5 sequences) subfamilies. The predominant major IE protein species, which are the subject of this review, are highlighted in blue. It is uncertain whether the IE1 isoforms shown in gray are present in CMV-infected cells [4]. All IE proteins are expressed from differentially spliced mRNAs, but exon 5 also encodes at least two different late proteins whose mRNAs are transcribed from internal promoters (LP). None of the minor IE1 and IE2 protein isoforms has been characterized with respect to activities in intrinsic or innate immune evasion.

Within the >230,000 base pair CMV DNA genome, the major IE gene is believed to have a decisive role in acute infection and reactivation from viral latency. Through differential splicing, polyadenylation and promoter usage this viral genomic region produces multiple mRNAs (reviewed in [3]). Although a variety of protein products expressed from these mRNAs have been identified [4–7] (Figure 1), the 72-kDa IE1 and the 86-kDa IE2 nuclear phosphoproteins are the most abundant and important. They share 85 amino-terminal amino acids corresponding to major IE exons 2 and 3 but have distinct carboxy-terminal parts encoded by exon 4 (IE1) or exon 5 (IE2) (Figure 1). While IE2 is absolutely indispensable for productive viral replication [8,9], IE1 is only conditionally essential. In fact, IE1-null viruses replicate efficiently in fibroblasts at a high multiplicity of infection (MOI). However, the absence of IE1 results in severely attenuated viral replication under low MOI conditions [10,11].

### 2.2. Functional Activities of the IE1 and IE2 Proteins

IE2 is the principal transcriptional activator of the CMV early genes [8,9]. In addition, IE2 negatively regulates viral gene expression including its own transcription (reviewed in [3,12]). IE1 appears to synergize with IE2 to promote transcriptional activation of the viral early genes, at least after low MOI infection [10,13]. Moreover, both IE1 and IE2 have been shown to activate certain host cell promoters (e.g., [6,14–20]) (reviewed in [3,12]). More recently, it has also been demonstrated that the two major IE proteins can individually block induction of distinct sets of potentially antiviral host genes [21–25]. In IE1, this activity depends at least partially on interactions with human signal transducer and activator of transcription (STAT) proteins. Beyond that, transcriptional regulation by IE1 and IE2 appears to involve multiple interactions with basal and accessory cellular transcription factors (reviewed in [1,3,12]) including histone modifying enzymes [24,26–30]. The latter are important for IE1- and IE2-dependent regulation of core histone tail acetylation and/or methylation [26–28,31]. In addition to their effects on the covalent modification and function of chromatin-associated proteins, IE1 and IE2 may also more directly act on DNA metabolism and structure. IE2 has been shown to block host cell DNA replication [32–34]. Furthermore, both major IE proteins seem to introduce mutations in cellular DNA [35], although the relevance of this observation remains to be determined. The chromatin-based activities of IE1 and IE2 are linked to their intranuclear localizations in ways that have not been fully elucidated. While IE2 binds sequence-specifically to DNA [36], IE1 does not seem to interact with DNA directly. However, IE1 associates with (mitotic) host cell chromatin [37,38], presumably via protein-protein interaction. In addition, both IE1 and IE2 target to interchromatinic matrix-associated nuclear domain 10 (ND10) compartments of the cell nucleus. IE1 disrupts these compartments most likely via interaction with the promyelocytic leukemia (PML) protein, the main structural organizer of ND10 [39–42].

Other activities associated with IE1 and IE2 expression concern effects on cell cycle progression and cell survival (reviewed in [43]). Ectopic IE1 can trigger a p53-dependent G1 growth arrest response [44], while it induces quiescent cells to enter S phase in p53-negative cells [45]. IE1 might stimulate S phase entry via interaction with the pocket protein p107 [46–48] and/or phosphorylation of p107, p130, and E2Fs through a reported kinase activity [49]. Similarly, transient IE2 expression may either promote cell cycle progression to the G1/S interface or growth arrest at G1/S or G2/M [33,44,50–52] (reviewed in [53]). IE2 may even induce premature cellular senescence [54]. At least some of the IE2-dependent effects on the cell cycle likely involve interactions with p53 and/or the retinoblastoma (Rb) tumor suppressor protein [55–59]. Finally, both IE1 and IE2 have been shown to counteract apoptotic cell death [60–65].

A comparative summary of activities that have been ascribed to the CMV IE1 and IE2 proteins is presented as Table 1.

### 2.3. IE1 and IE2 in Innate and Intrinsic Immunity

The innate (in the broader sense) immune system of many organisms includes both inducible (innate in the stricter sense) and constitutive (intrinsic) mechanisms. Inducible innate mechanisms largely depend on pathogen-initiated cytokine production and signaling. Intrinsic responses like apoptosis, autophagy, and viral genome-directed repression mechanisms have only recently been recognized as an essential component of immunity which gives all cells the capacity to respond to infection. In the following paragraphs we will expand on the activities of CMV IE1 and IE2 that have established the two viral proteins as antagonists of innate and intrinsic antiviral host responses.

## 3. The CMV IE1 and IE2 Proteins Exhibit Antiapoptotic Potential

### 3.1. Apoptosis Pathways in Antiviral Host Defense

Apoptosis is a genetically regulated process of cell suicide resulting from activation of intrinsic or extrinsic death signaling pathways (reviewed in [67,68]). Intrinsic proapoptotic signals generated by diverse stresses such as growth factor withdrawal, DNA damage, or hypoxia trigger mitochondrial membrane permeabilization. Permeability transition is linked to mitochondrial dysfunction and the release of cytochrome c and other proapoptotic factors from the organelle’s intermembrane space into the cytosol. These factors eventually activate “initiator” cysteine aspartase (caspase) 9 as well as caspase-independent downstream mechanisms [67]. The control of mitochondrial apoptotic events is largely accomplished through the Bcl-2 family of proteins, which include both pro- (e.g., Bax) and antiapoptotic (e.g., Bcl-2) members (reviewed in [69]). The tumor suppressor protein p53, in turn, has a critical role in regulating the expression of Bax and other key proteins involved in (intrinsic) apoptosis (reviewed in [70]). The extrinsic signaling pathways that initiate apoptosis start with “death receptor” activation at the cell surface. For example, tumor necrosis factor (TNF) α or Fas ligand bind to TNF receptor 1 or Fas/CD95, respectively (reviewed in [71]). These interactions induce receptor oligomerisation, recruitment of cytoplasmic adapter proteins, and formation of a death-inducing signaling complex with initiator caspase-8. The complex subsequently triggers activation of caspase-8, which is negatively regulated by the FLICE inhibitory protein (FLIP). For most cell types, extrinsic signals are also amplified by crosstalk with the intrinsic mitochondrial pathway and caspase-9 activation. Intrinsic and extrinsic pathways eventually converge on a downstream “execution phase”. Caspase-3 is considered to be the most important effector or “executioner” caspase and is activated by any of the initiator caspases. Executioner caspases cleave various protein substrates ultimately causing the biochemical and morphological hallmarks of apoptotic cells including chromatin fragmentation and cellular disintegration.

Apoptosis typically occurs during development and as a homeostatic mechanism in normal cell turnover. However, the elimination of infected cells via apoptotic cell death is also considered one of the most primordial defense mechanisms against intracellular pathogens including viruses. Thus, disabling host cell apoptosis might represent an obligate step in the viral life cycle. Correspondingly, numerous viral proteins have been reported to modulate the apoptotic response of the host cell to infection (reviewed in [72]).

### 3.2. Role of IE1 and IE2 in Apoptosis Inhibition

Although it is now clear that cytomegaloviruses employ multiple strategies to delay cell death in infected cells (reviewed in [73]), the IE1 and IE2 proteins were the first CMV gene products reported to block apoptosis [60]. Following transient or stable expression in HeLa cells, IE1 and IE2 individually inhibited induction of extrinsic apoptosis by short exposure to TNF-α and cycloheximide or by infection with a proapoptotic mutant (E1B-19kDa-deficient) adenovirus. However, the viral proteins did not protect HeLa cells from TNF-α-or Fas-mediated apoptosis under more stringent experimental conditions [74] or when cell death was triggered by irradiation with ultraviolet (UV) light [60]. Subsequently, the anti-apoptotic effects of ectopic IE1 and/or IE2 expression have been confirmed in other cancer cell lines and in primary cells [28,61–64,75]. However, the impact of the IE1- and IE2-associated pro-life activities on CMV infection remain unexplored.

There is no evidence that IE1 or IE2 interfere with mitochondria-related apoptotic processes, and the two proteins do not alter the expression of Bcl-2 or Bax [60]. Instead, IE1 and IE2, expressed in concert or individually, inhibit apoptosis by activating the phosphatidylinositide 3′-OH kinase (PI3K) pro-survival pathway [61,62], which is also induced by CMV infection [62,76,77]. This has been demonstrated in the *ts13* cell line, which carries a temperature-sensitive allele of the gene encoding TAF_II_250 and therefore undergoes apoptosis at the non-permissive temperature. Furthermore, combined expression of the viral IE proteins increased the activity of the serine/threonine kinase Akt (also known as protein kinase B), a major PI3K downstream target (reviewed in [78,79]). Akt promotes cell survival in part by targeting IκB kinase, which phoshorylates IκB resulting in nuclear localization of NFκB and activation of NFκB-responsive promoters of antiapoptotic genes. In fact, several cellular [15–17], viral [80–82] and artificial [62] promoters have been shown to be transcriptionally activated by CMV IE1 in an NFκB-dependent fashion. However, IE2 may rather repress than stimulate transcription from NFκB-regulated promoters [21,22,25,83]. On the other hand, IE2 appears to activate expression of cellular FLIP in CMV-infected retinal pigment epithelial cells and human retina tissue [65]. FLIP blocks the apoptotic pathway by interacting with caspase-8 at the death-inducing signaling complex. Notably, IE2-specific up-regulation of FLIP in CMV-infected retinal cells depends on PI3K [65].

In addition, mechanisms involving the tumor suppressor protein p53 have been proposed to account for the observed inhibitory effects of CMV IE2 on cellular apoptosis. IE2 binds to p53 and interferes with the tumor suppressor protein’s transcriptional activator function [55,56,84]. It was further demonstrated that IE2 can repress the acetylase activity of p300/cAMP response element binding protein binding protein (CBP) towards p53, rendering the tumor suppressor protein unable to execute UV-dependent apoptosis of colon cancer cells [28]. Moreover, expression of IE2, but not IE1, protects smooth muscle cells from p53-mediated apoptosis [63]. Rather than p53, IE1 targets the tumor suppressor protein PML [41], but the functional impact of this interaction on cell survival has not been evaluated. This potential link warrants future investigation since PML is known to affect PI3K signaling, p53 activity, and apoptosis (reviewed in [85,86]).

In summary, it appears that each of the CMV major IE proteins can block extrinsic apoptosis pathways via activation of PI3K signaling, although no physical interaction partner (besides PML) of IE1 or IE2 has so far been identified in this pathway. Beyond that, additional mechanisms likely contribute to inhibition of apoptosis by the viral proteins that may involve IE2-p53 complex formation and other known or unidentified interactions. Despite the fact that the antiapoptotic potential of the two major IE proteins has clearly been established in several overexpression settings, its true relevance for viral infection and pathogenesis remains to be determined.

## 4. The CMV IE1 Protein Counteracts ND10-Dependent Antiviral Responses

### 4.1. Association of Parental Viral Genomes and IE Proteins with ND10

A general feature of nuclear replicating DNA viruses including CMV is the preferential association of their parental genomes and prereplicative sites with functionally promiscuous interchromatin protein complexes known as ND10 (reviewed in [87] and in the article by Tavalai and Stamminger in this issue). Viral genome deposition at ND10 is followed by targeting of the *de novo* synthesized IE1 and IE2 proteins to these subnuclear complexes [42,88]. While IE1 usually colocalizes precisely with all nuclear ND10, IE2 was shown to switch between perfectly overlapping and juxtaposed locations relative to a subset of these structures [89]. In fact, it has been proposed that IE2 foci and ND10 represent separate complexes that form independently during infection [89]. In any case, the spatial interplay between IE2 dots and ND10 can only be observed within a short time interval due to the action of the IE1 protein. IE1 disrupts ND10 during the early phase of CMV infection and upon ectopic expression [39,40,42]. The exact mechanism of IE1-dependent ND10 disruption remains unclear, although it likely involves binding to and de-SUMOylation of the PML protein [41,90,91].

### 4.2. IE1 as Antagonist of ND10-Related Intrinsic Defenses

PML is a major constituent of ND10, and two main lines of evidence support the idea that this protein mediates an intrinsic immune response against CMV. First, the course of the CMV infectious cycle is significantly attenuated in cells overexpressing PML [92]. Secondly, short interfering RNA (siRNA)-mediated depletion of PML results in markedly increased IE gene expression and more efficient initiation of productive infection [93]. Importantly, PML knock-down efficiently compensates for IE1 in promoting replication of an IE1-deficient mutant virus [93]. This observation extends earlier findings suggesting a link between ND10 disruption and the activities of IE1 in transcriptional activation of viral early gene expression [92].

Interestingly, not only PML but also other ND10 components including death domain-associated protein (Daxx) and α-thalassemia/mental retardation syndrome X-linked protein (ATRX) appear to be involved in an intrinsic antiviral response against CMV that limits viral gene expression [93–97]. Several of these ND10 proteins have been implicated in epigenetic processes (reviewed in [98]). For example, Daxx interacts with histones as well as histone deacetylases (HDACs) and forms part of a chromatin remodelling complex together with ATRX [99,100]. Therefore, it is tempting to speculate that ND10 proteins may contribute to formation of a repressive chromatin structure on newly infecting virus DNA. In fact, evidence for a “pre-IE” intrinsic antiviral response based on hypoacetylation and repressive methylation of core histones associated with CMV genomes has very recently been provided by Groves *et al.* [101]. In this context it is relevant to note that the CMV (and murine cytomegalovirus) IE1 protein is known to promote histone acetylation, at least in part by antagonizing HDAC activity [26,102]. It should also be mentioned that the CMV tegument protein pp71 interacts with Daxx and antagonizes Daxx-mediated histone deacetylation to activate the viral major IE promoter by targeting the cellular repressor protein for degradation [95,132–134]. Futhermore, pp71 displaces ATRX from ND10 at pre-IE times post CMV infection [97]. These observations suggest that pp71 may contribute to relieving ND10-based intrinsic defenses even prior to IE gene expression.

Nonetheless, it seems likely that counteracting intrinsic repression mediated by ND10 or at least one of its components (*i.e.*, PML) is a key activity by which CMV IE1 facilitates productive viral replication at low viral input multiplicities (assumed to be the natural mode of infection). The IE1-dependent effects on histone acetylation and ND10 integrity may both contribute, either individually or interdependently, to evasion from ND10-related antiviral defenses.

## 5. The CMV IE1 and IE2 Proteins Counteract Antiviral Cytokine Responses

### 5.1. Cytokine-Based Innate Immunity Against Viral Infection

Cytokines are secreted proteins which mediate fundamental processes in immune control and inflammation. Certain cytokines, such as TNF-α and interferons (IFNs), produce intracellular signals that can cause apoptotic, cytostatic, or directly antiviral processes thereby limiting virus replication (reviewed in [103–105]). Particularly type I IFNs (primarily IFN-β and multiple subtypes of IFN-α) contribute critically to the induction of the innate immune response against most if not all viruses including CMV (reviewed in [103,104,106,107]).

Expression of type I IFNs results from stimulation of pattern recognition receptors and is transcriptionally regulated through coordinated activation of latent (*i.e.*, inactive) transcription factors including (among others) NFκB and IFN regulatory factor 3 (IRF3). Following gene induction and protein secretion, type I IFNs bind to their cognate cell surface receptor, resulting in phosphorylation of tyrosine kinase 2 (Tyk2) and Janus kinase 1 (Jak1). The activated kinases subsequently phosphorylate the STAT1 and STAT2 proteins. This leads to heterodimerization of the STATs, association with IFN regulatory factor 9 (IRF9), and nuclear translocation of the trimeric complex (termed IFN-stimulated gene factor 3, ISGF3) (reviewed in [108]). Although this classical paradigm of Jak-STAT signal transduction has recently been challenged (reviewed in [109]), there is general agreement that ISGF3 ultimately binds sequence-specifically to promoters of numerous IFN-stimulated genes (ISGs), resulting in their transcriptional activation. ISG products are the terminal IFN effector molecules many of which contribute to either inhibiting virus replication in infected cells or to establishing an “antiviral state” in uninfected cells. Examples of typical ISGs with antiviral activity include ISG15, MxA, PML, protein kinase R, and 2′,5′-oligoadenylate synthetase (reviewed in [103,108]). Type I IFNs are also known to help activating natural killer (NK) cells and, although IFN-γ (type II IFN) plays a major role in promoting transition from innate to adaptive immune responses, IFN-α/β are also important in this regard. For instance, type I IFNs promote the maturation of dendritic cells and sustain the proliferation of antigen-specific CD8^+^ T cells. Moreover, these cytokines upregulate expression of class I major histocompatibility complex (MHC) molecules and other components of the antigen presenting machinery (reviewed in [103,110]).

Despite their antiviral potential, CMV infection activates IRF3 and NFκB inducing ISGs and type I IFNs (reviewed in [106,107]). However, infections performed in the absence of viral gene expression induce these genes more strongly [22, 111,112]. Yet even UV-inactivated virus did not seem to trigger full activation of the type I IFN pathway [111,113]. These observations suggested that CMV limits the type I IFN response through the actions of viral products introduced with the virus particle and synthesized during infection. Indeed, subsequent work has demonstrated that CMV interferes with multiple distinct steps in type I IFN synthesis, IFN-dependent signaling, and ISG effector function via both viral tegument and IE proteins [22–24,114–120]. Besides the IFN response, CMV also attenuates interleukin (IL) 1β and TNF-α proinflammatory signaling [121,122]. The CMV antagonists of proinflammatory cytokine (including type I IFN) induction and signal transduction include the IE1 and IE2 proteins.

Proinflammatory cytokines and type I IFNs are also known to induce the expression of several chemokines. Chemokines are cytokines with selective chemoattractant properties coordinating the homeostatic circulation of leukocytes as well as their movement to sites of inflammation or injury (reviewed in [123]). Chemokines are either divided into four subfamilies (C, CC, CXC, and CX3C), based on the arrangement of conserved amino-terminal cysteine residues, or into homeostatic and inflammatory proteins based on their patterns of expression and associated function. Homeostatic chemokines are constitutively produced and primarily involved in maintaining normal leukocyte trafficking. In contrast, expression of inflammatory chemokines (e.g., IL-8, MIP-1a, and RANTES) is typically induced in activated cells, and these proteins recruit leukocytes to inflamed tissues. In fact, chemokines attract the first wave of innate immune cells, including neutrophils, monocytes, and NK cells, all of which express inflammatory chemokine receptors (G protein-coupled receptors through which chemokines exert their function). Chemokines also recruit dendritic cells, which provide the link between innate and adaptive immunity, and stimulate the effector mechanisms of lymphocytes. Consequently, chemokines play a pivotal role in the resolution of virus infections (reviewed in [123]).

As in the case of type I IFNs, CMV infection first activates the production of multiple chemokines, such as RANTES and MCP-1, in endothelial cells [124,125], fibroblasts [111,125,126], and monocytes [127]. However, CMV has also evolved multiple strategies to counteract chemokine-mediated immune responses, including sequestration by virus-encoded chemokine receptors [124,128,129] and direct suppression of chemokine production [124,130]. The latter process involves the viral IE2 protein.

### 5.2. IE2-Mediated Inhibition of Cytokine Induction

The CMV IE2 protein can efficiently block virus-induced expression of (inflammatory) chemokines including IL-8, MCP-2, MIG, MIP-1a, and RANTES [25]. Moreover, IE2 inhibits induction of the cytokines IFN-β [22] and IL-6 [21,83] and of genes responsive to TNF-α [21]. Since most of these genes carry NFκB binding sites in their promoters, it was subsequently examined whether IE2 interferes with the function of this transcription factor. In fact, IE2 appears to target intranuclear NFκB to prevent or reverse virus- and TNF-α-induced sequence-specific DNA binding by this transcription factor. Interestingly, IE2 also blocks the activity of NFκB when the activation domain of the cellular transcription factor is artificially tethered to a promoter [83]. This may indicate that IE2 inhibits NFκB-dependent transcriptional activation at a step subsequent to promoter recruitment.

### 5.3. IE1-Mediated Inhibition of Jak-STAT signaling

The ability to inhibit potentially antiviral cytokine and chemokine induction does not seem to be shared between the CMV IE2 and IE1 proteins. In particular, CMV-induced IFN-β gene epxression was not detectably counteracted by IE1 in various different settings [22–24]. However, IE1 has been shown to antagonize the type I IFN response at a step downstream of IFN production and secretion by interfering with Jak-STAT signaling.

**Figure 2.**
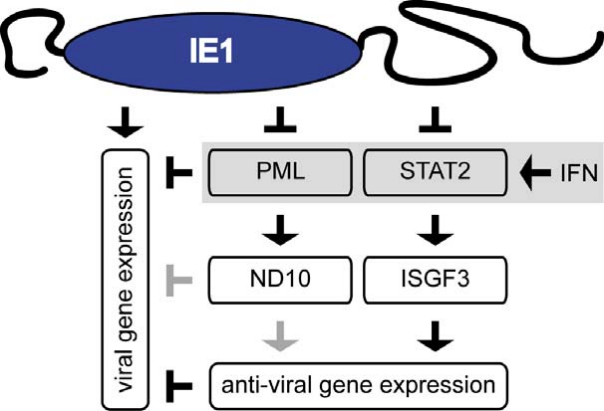
Model of IE1 activities in inhibition of IFN-mediated antiviral gene expression and induction of viral gene expression. The predicted structural organization of CMV IE1, comprising a central globular domain and natively unstructured regions at the protein termini [131], is depicted at the top of the diagram. IE1 is proposed to antagonize the type I IFN-mediated antiviral host cell response by at least two distinct mechanisms. The first mechanism depends on binding of IFN-activatable STAT2 to the carboxy-terminal IE1 domain resulting in inhibition of ISGF3-mediated anti-viral gene induction. The second mechanism is independent of IE1-STAT2 complex formation and may involve a previously described interaction with the IFN-inducible PML protein resulting in disruption of ND10, which has been mapped to central parts of the viral protein. Impaired PML function and ND10 integrity have both been linked to derepression of viral gene expression. In addition, ND10 may be involved in regulation of antiviral gene expression. Note that IE1 has also been proposed to activate viral gene expression more directly via interaction with cellular transcription factors and at least one HDAC. Black symbols represent experimentally verified connections, and gray symbols indicate hypothetical links.

IE1 does not detectably interfere with the expression, stability, phosphorylation, or nuclear translocation of the STAT1 and STAT2 proteins. However, IE1 forms stable complexes with both STATs inside the nuclei of transfected and CMV-infected cells [23,24]. Moreover, the viral protein can redirect STAT2 (and STAT1) to nuclear matrix-associated (ND10) and chromatin compartments. Similar to what has been described for IE2 regarding NFκB targeting, IE1 precludes sequence-specific promoter binding by all three components (STAT1, STAT2, and IRF9) of ISGF3 [23,24]. However, STAT2 appears to be the viral protein’s primary target in the trimeric complex (S. Krauss, S. Meinel, I. Tschertner, C. Paulus, and M. Nevels, unpublished results). Interestingly, IE1-STAT2 complex formation appears to be negatively regulated by SUMOylation of the viral binding partner [24]. In accordance with reduced ISGF3 DNA binding, induction of ISGs including MxA, ISG54, and CXCL10 by CMV infection (or exogenous IFN treatment) is substantially dampened in the presence of IE1 [23,24]. Through STAT2 interaction the viral protein ultimately confers a substantial degree of protection against the antiviral effects of IFN-α and IFN-β upon CMV [23,24,131]. However, IE1 counteracts the antiviral type I IFN response and promotes viral replication by at least two distinct mechanisms, one depending on sequestration of STAT2 and the other one likely involving ND10 interaction [131] (Figure 2).

## 6. Conclusions

The innate host cell response to viral infection is in large parts characterized by the induction of cytokines and inflammatory chemokines. In addition, constitutively active intrinsic antiviral defense mechanisms (e.g., apoptosis and chromatin-based repression) ubiquitously exist. The IE1 and IE2 proteins are among the first *de novo* synthesized proteins following primary CMV infection or viral reactivation. It emerges that a principal task of IE1 and IE2 is to counteract intrinsic and innate host responses that would otherwise terminate the viral life cycle in its very beginnings. In particular, IE1 antagonizes apoptosis, ND10-related transcription silencing, and type I IFN signaling. Likewise, IE2 inhibits apoptosis and inflammatory cytokine/chemokine induction. In comparison to the long recognized direct effects of IE1 and IE2 on viral transcription, their rather recently discovered immune evasion activities may turn out to be equally important to assure viral replicative success. Consequently, the interactions of IE1/IE2 with host cell innate and intrinsic defense pathways may provide new opportunities for antiviral intervention.

## Figures and Tables

**Table 1.** Common and distinct activities of the CMV IE1 and IE2 proteins.

**Activities**	**IE1**	**IE2**	**Selected References**
**CMV replication**			

Requirement for viral replication at low MOI	+	+	[8–11]
Requirement for viral replication at high MOI	–	+	[8–11]
**Cell cycle and apoptosis**			

Inhibition of cell cycle progression	+	+	[33, 44,50–52]
Induction of cell cycle progression	+	+	[33, 45,51,66]
Inhibition of apoptosis	+	+	[60–65]
Induction of senescence	–	+	[54]
**Nuclear structures**			

ND10 targeting	+	+	[39,40,42]
ND10 disruption	+	–	[39,40,42]
Mitotic chromatin association	+	–	[37,38]
DNA binding	–	+	[36]
**Histone modification**			

Core histone tail acetylation	+	(+)	[26]
Core histone tail methylation	–	+	[27,31]
**DNA metabolism**			

Inhibition of cellular DNA replication	–	+	[32–34]
Induction of mutations in cellular DNA	+	+	[35]
**Transcription**			

Activation of viral genes	+	+	reviewed in [1,3,12]
Repression of viral genes	–	+	reviewed in [1,3,12]
Activation of cellular genes	+	+	[6,14–20]
Inhibition of cellular gene activation	+	+	[21–25]
**Other activities**			

Kinase activity	+	–	[49]

+, positive;

(+), likely positive;

–, negative or unknown. Shading indicates activities most relevant to this review.
